# Derivation of clinical prediction rules for identifying patients with non-acute low back pain who respond best to a lumbar stabilization exercise program at post-treatment and six-month follow-up

**DOI:** 10.1371/journal.pone.0265970

**Published:** 2022-04-27

**Authors:** Christian Larivière, Khalil Rabhi, Richard Preuss, Marie-France Coutu, Nicolas Roy, Sharon M. Henry

**Affiliations:** 1 Institut de recherche Robert-Sauvé en santé et en sécurité du travail (IRSST), Montréal, Québec, Canada; 2 Center for Interdisciplinary Research in Rehabilitation of Greater Montreal (CRIR), Institut Universitaire sur la Réadaptation en Déficience Physique de Montréal (IURDPM), Centre Intégré Universitaire de Santé et de Services Sociaux du Centre-Sud-de-l’Ile-de-Montréal (CCSMTL), Montréal, Québec, Canada; 3 Independent Statistician Consultant, Montréal, Québec, Canada; 4 School of Physical & Occupational Therapy, McGill University, Montréal, Québec, Canada; 5 Charles-Le Moyne Hospital Research Centre, Université de Sherbrooke, Longueuil, Quebec, Canada; 6 Department of Neurological Sciences, University of Vermont, Burlington, Vermont, United States of America; Mugla Sitki Kocman Universitesi, TURKEY

## Abstract

Low back pain (LBP) remains one of the most common and incapacitating health conditions worldwide. Clinical guidelines recommend exercise programs after the acute phase, but clinical effects are modest when assessed at a population level. Research needs to determine who is likely to benefit from specific exercise interventions, based on clinical presentation. This study aimed to derive clinical prediction rules (CPRs) for treatment success, using a lumbar stabilization exercise program (LSEP), at the end of treatment and at six-month follow-up. The eight-week LSEP, including clinical sessions and home exercises, was completed by 110 participants with non-acute LBP, with 100 retained at the six-month follow-up. Physical (lumbar segmental instability, motor control impairments, posture and range of motion, trunk muscle endurance and physical performance tests) and psychological (related to fear-avoidance and home-exercise adherence) measures were collected at a baseline clinical exam. Multivariate logistic regression models were used to predict clinical success, as defined by ≥50% decrease in the Oswestry Disability Index. CPRs were derived for success at program completion (T8) and six-month follow-up (T34), negotiating between predictive ability and clinical usability. The chosen CPRs contained four (T8) and three (T34) clinical tests, all theoretically related to spinal instability, making these CPRs specific to the treatment provided (LSEP). The chosen CPRs provided a positive likelihood ratio of 17.9 (T8) and 8.2 (T34), when two or more tests were positive. When applying these CPRs, the probability of treatment success rose from 49% to 96% at T8 and from 53% to 92% at T34. These results support the further development of these CPRs by proceeding to the validation stage.

## Introduction

Low back pain (LBP) remains one of the most incapacitating health conditions worldwide, and one for which the majority of suffers cannot be given a reliable diagnosis of the cause(s) of their pain [1]. This dilemma often leads to the use of generic treatments, such as exercise programs that do not account for individual patient presentation, leading, at best, to modest population-level clinical effects. Although some exercise approaches, such as strength/resistance and coordination/stabilization training, generally lead to greater clinical effects than other exercise approaches (e.g., aerobic exercise or combined programs) [2], these effects also remain modest. Further research is needed to develop clinical prediction rules (CPRs) for treatment success, effectively identifying subgroups of LBP patients that are likely to respond to specific exercise approaches.

Lumbar stabilization exercise programs (LSEP) target motor control and coordination of the paraspinal and abdominal musculature, and may also progressively overload muscles to enhance trunk muscle endurance [3–5]. This approach should theoretically be beneficial to patients showing clinical lumbar instability [6] and is particularly attractive from various perspectives. LSEP have been shown to promote neuroplastic changes in the motor cortex, along with associated changes in muscular coordination [7]. They also encompass psychological principles relevant to chronic pain, with gradual exposure to physical activity, allowing a reduction of fears associated with pain and movement [8]. LSEP are also consistent with strategies known to maximize clinical outcomes, namely supervised active exercise at high dosage [9, 10]. While the latter generally requires a home exercise program, this is feasible if it does not require specialized equipment. It is also consistent with the goal of promoting patient self-efficacy [11].

Another research group has published preliminary work, at the derivation stage, on the development of CPRs for an LSEP [12]. The construct validity of that CPR was tested successfully [13, 14], but its formal validation was unsuccessful due to a lack of statistical power [15]. While these results show that it is possible to derive, and at least partially validate, a predictive CPR for LSEP success, this derivation [12] may have suffered from limitations other than the size of the preliminary cohort (n = 54 patients). First, the patient sample was heterogeneous in terms of symptom duration (acute, subacute, and chronic phases), even though physical exercises are generally not effective during the acute phase of LBP [16]. Second, a limited number of clinical tests to assess for functional lumbar segmental instability (LSI) and motor control impairment (MCI) of the pelvic-lumbar area [17] were included. Third, only one psychological variable (fear-avoidance beliefs from physical activity) was taken into consideration. Other variables from the psychological model of fear-avoidance (e.g. pain catastrophizing, psychological distress/depression) also warrant consideration [8], along with variables that could be predictive by their virtue of their effect on exercise adherence (particularly for home exercises). Fourth, the exercise program was generally consistent with an isometric endurance/strengthening program (often called the McGill approach) [4]. Clinicians, however, often also incorporate exercises focusing purely on motor control (often called the Australian approach) [5], at least early on. Finally, the previously derived CPRs only predicted success at the end of treatment, whereas a prediction of longer-term success would also be desirable [18].

The aim of the present study was to perform the derivation stage of CPRs for treatment success using an LSEP, for adult patients with non-acute low back pain. The LSEP was a hybrid between the McGill and Australian approaches, using exercises specific to motor control and to isometric endurance/strengthening. The primary goal was to extend previous positive findings [12] by using a more comprehensive clinical investigation, including more physical and psychological measures applied to a larger sample of patients. Moreover, success was predicted at the end of treatment as well as at six-month follow-up.

## Materials and methods

### Design of the study

**Fig 1:**
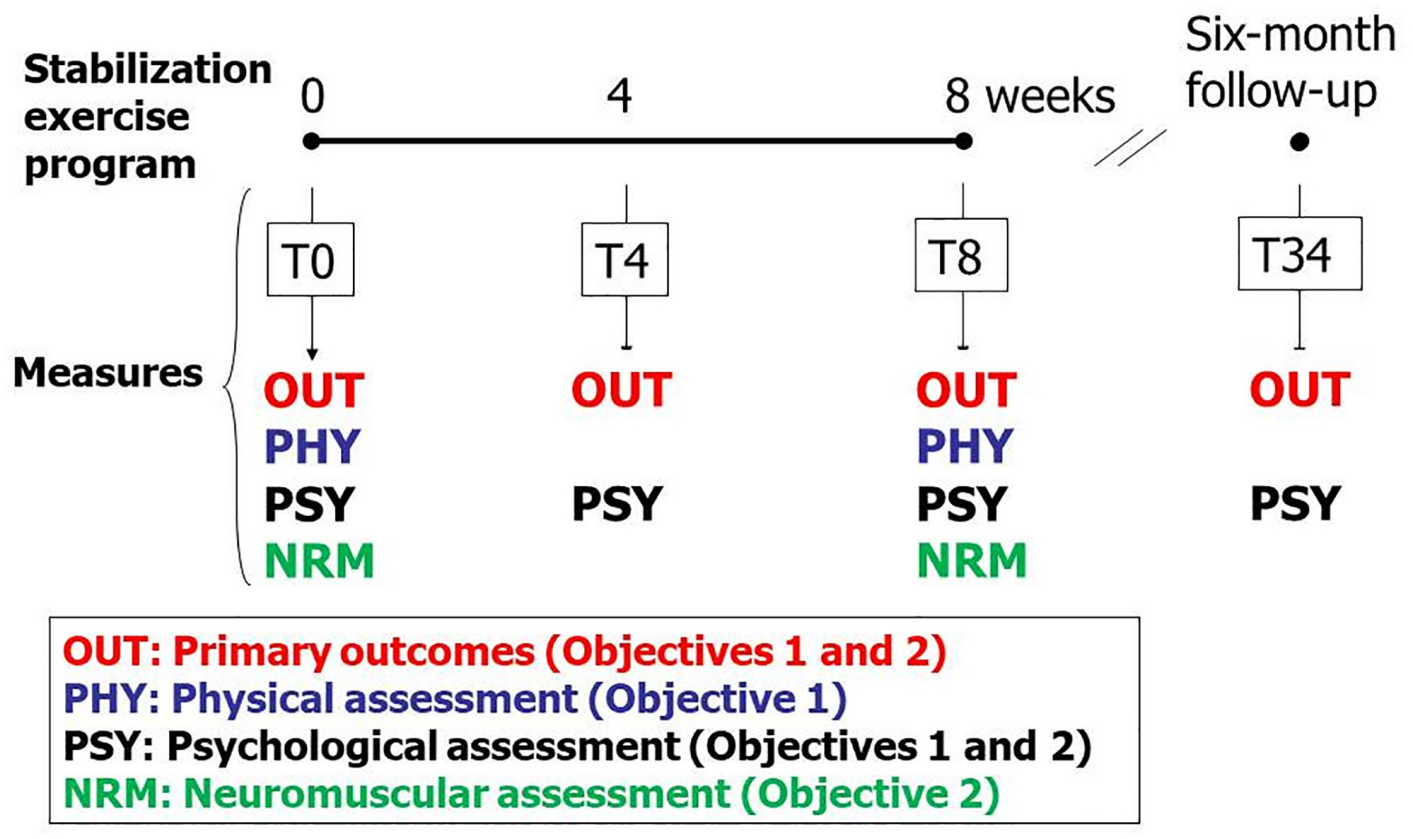
Four categories of measures (OUT, PHY, PSY, NRM) were collected at different times during (T0, T4, T8) and following (T34) the lumbar stabilization exercise program. PHY and NRM measures are more burdensome and were consequently only collected at T0 and T8.

A prospective one-arm study involving three assessments taken during the intervention and a six-month follow-up was carried out in a single patient group (Fig 1). While some controversy exists as to the use of a one-arm or a two-arm (RCT) design for the derivation of a CPR [19, 20], all CPR to date have used a one-arm design at the derivation stage [20]. As such we have also elected to use a one-arm design. To reduce the likelihood of spurious findings, the potential variables were selected according to a sound theoretical rationale using well-known theoretical models. There were still, however, too many variables to conduct a RCT with sufficient power for interaction testing (resource problem). Thus, a RCT should be conducted later for the validation stage.

This study design allows us to pursue two objectives, namely to 1. to derive two CPRs (treatment success at the end of treatment and at six-month follow-up) using practical (clinical) measures obtained during the initial clinical examination and 2. to identify the mechanisms involved in this exercise program through an expanded battery of measures, thus allowing the specific effects of the treatment to be described. The present study will address the first objective (CPRs’ derivation), using physical (PHY) and psychological (PSY) measures collected at T0 and primary clinical outcome measures (OUT) collected at T0, T8 and T34 (Fig 1). The other findings derived from this study design will be published elsewhere.

### Participants

Using preliminary findings [21] derived from 64 participants distributed in four subgroups (defined in section 3.5)–success (n = 31), failure (n = 12), improvement (n = 5), dropouts (n = 16)—the sample size required to finalize the CPR derivation stage was estimated using two methods. These estimations were performed with the data corresponding to the preliminary CPR at T8. In the first method, an a priori power analysis was performed using GPower 3.1 program [22], using the procedure proposed by Demidenko [23] for a sample size estimation for logistic regression, as it is considered more accurate than others [24, 25]. Effect sizes (odds ratios) considered for the CPR for success and failure were 13.7 (95%CI 2.0–94.0) and 19.4 (95%CI 2.4–157.0), respectively. By adjusting for the other covariates in the model, considering an alpha of .05, a power of .80 and an attrition rate of 25%, we calculated an estimated sample size of 93 participants. The second method simply considers that the number of participants in subgroups will be uneven. Using the ratio between the subgroup numbers from the preliminary data, we calculated an estimated sample size of 107 participants. The more conservative scenario of 107 participants was retained as our minimum sample size (allowing for 25% attrition).

Participants were recruited through newspaper advertisements and from physiotherapy clinics in Montreal, Quebec, Canada, and were assessed from July 2012 to August 2016 (preliminary study or phase 1) and from July 2018 to October 2020 (phase 2). They were French- or English-speaking, aged between 18 and 65 years, had lumbar or lumbosacral pain, with or without radicular pain, for at least four weeks (non-acute phase) and a minimum score of 12% on the Oswestry Disability Index (ODI) [26], thus allowing a minimal clinically important change of 10% [27] to occur. Participants were selected in the non-acute phase because exercise is generally considered to be more effective after the acute phase of LBP [28, 29]. Exclusion criteria were the initiation of an exercise program within the last six months; thoracic or neck pain that is more severe than LBP; a specific lumbar pathology (fracture, infection or tumor) or scoliosis; surgery on the pelvis or spinal column; systemic or degenerative disease; pregnancy; litigation relative to the back injury; and the presence of one positive neurological sign in two of three test categories: (a) reduced Achilles and patellar tendon reflexes, (b) reduced strength in lumbosacral myotomes, (c) reduced sensation in lumbosacral dermatomes. Some care was taken to balance the sex of participants across age strata.

All procedures and the informed written consent were approved by the ethics committees of the Centre for Interdisciplinary Research in Rehabilitation of Greater Montreal (CRIR) (ethical registration numbers: CRIR-738-0512 and CRIR-1315-0318 for preliminary and final studies, respectively).

### Lumbar Stabilization Exercise Program (LSEP)

The 8-week LSEP was implemented by 15 physiotherapists from private physiotherapy clinics in the Montreal area, without any co-interventions (e.g., occupational therapy, TENS, massage, ultrasound), except for medications (non-steroidal anti-inflammatory drugs, analgesics, opioids, muscle relaxants). In order to standardize the information given to patients on their condition, the Back Book booklet [30], or its French translation (*Guide du dos*: ISBN: 978-2-923465-03-6), was given to patients at their first clinical visit. The booklet aimed to change beliefs and behaviours (resuming activities) related to back pain. Physiotherapists received a one-day training session explaining how to describe the study (1 hour) and, more importantly, how to teach patients the exercises (8 hours).

The exercise program, which has been previously detailed ([31]; see Additional file 1), was essentially consistent with initial use of the Australian approach [5], which focuses on motor control of the deep trunk muscles, followed by the McGill approach [4], which includes overload exercises targeting trunk muscle endurance (rather than strength). It consisted of three phases: 1. pain management, including isolated contractions of the transversus abdominis (TrA) and lumbar multifidus (LuM), followed by progressive introduction of TrA/LuM co-contractions during low-load exercises (maximum two weeks); 2. beginning impairment and functional level, including exercises with an emphasis on quality of movement control; and 3. moderate/advanced impairment and functional level, with exercises focussed on muscle endurance (higher volume and intensity), while still co-contracting TrA and LuM.

The use of accessible feedback methods, such as palpation and the pressure feedback unit [5] (but excluding ultrasound imaging), was employed in this study in order to generalize our results to current physiotherapy practice. Physiotherapists adjusted the progression according to patient needs. All subjects completed the program in the clinic in two 30-minute sessions per week over an 8-week period (except for the first 60-minute visit for assessment by the assigned physiotherapist; no information was drawn from this assessment by the research team). During and after the 8-week program, patients were encouraged to exercise at home, using an exercise type and parameters sheet, according to the following schedule: 7, 3 and 3 days per week for Phases 1, 2 and 3 of the exercise program, respectively.

### Assessments

#### Primary Outcome Measures (OUT)

The ODI was used to assess LBP-related disability [26] while an 11-point (0 to 10) numeric pain rating scale (NPRS) was used to assess pain intensity [32]. More detailed information is provided in S1 File.

#### PHY-domain testing

The physical examination was conducted by a research-trained (M.Sc.) physical therapist (N.R.). The exam was comprised of tests that can be theoretically related to lumbar segmental instability (LSI) or motor control impairments (MCI) [17, 33, 34] and that have acceptable interrater reliability [kappa > 0.6; intraclass correlation coefficients—ICC > 0.70; [17]], as detailed in S1 File. PHY testing covered different dimensions, namely 1. LSI (n = 4), 2. MCI (n = 7), 3. posture and range of motion (ROM) (n = 6), 4. trunk muscle endurance (TME) (n = 4) and 5. physical performance tests (PPT) (n = 4). With respect to MCI tests, only symptoms caused by these tests were examined, because the skills required to evaluate signs (alignment, movements) are more complicated and less reliable [35]. For measures taken from both the left and right side (e.g., right and left lateral trunk flexion; left and right lower extremity measurements), and with the goal of retaining the measurements most associated with impairments, only the minimal ROM (exception: lateral trunk flexion) and TME scores, as well as the maximal scores during PPT (related to slow movements) and MCI tests were selected for further analyses.

#### PSY-domain testing

As detailed in S1 File, patient-reported outcome measures (PROMs) were collected. These included a brief screening tool (STarT Back) [36], variables from the fear-avoidance model (pain intensity, disability, pain catastrophizing, fear-avoidance beliefs, psychological distress, physical activity level), and variables theoretically related to home-exercise adherence [37–40]: pain during exercise, self-efficacy for exercise, anxiety, depression and helplessness, social or family support to exercise, illness perception, treatment/patient expectation for exercise and readiness to exercise. Concepts that could not be assessed before the second week of therapy, such as the patient/therapist alliance and treatment credibility and expectancies, although potentially predictive of home exercise adherence, could not be considered for the use in a CPR. Home-exercise adherence was assessed after the exercise program (T8) and at six-month follow-up (T34) to minimize the effect on patient behavior [41].

### Statistics

Three subgroups of patients were defined according to their level of success using ODI at T8 or T34: 1. success, 2. clinically significant improvement, and 3. failure, similar to Hicks, Fritz [12]. For this purpose, the ODI change score (e.g., ΔODI = ODIT0—ODIT8) and the corresponding percentage [e.g., ΔODI% = ((ODIT0—ODIT8) / ODIT0) × 100] were calculated for each patient. With the Oswestry questionnaire, a 50% improvement threshold has been used previously [12] and more recently justified as a valid criterion for defining clinical success in patients with low back pain [42]. A clinically important change of 10 points in ODI scores was also considered [27]. Using these criteria, the three subgroups were defined as follows:

- Success: ΔODI% ≥ 50
- Clinically significant improvement: ΔODI% < 50%, but ΔODI ≥ 10
- Failure: ΔODI% < 50% and ΔODI < 10

Logistic regression models were developed to define, on the basis of the initial measures (PHY and PSY measures at T0), the characteristics of the patients who had therapeutic success at T8 and then at T34.

The steps in these processes are briefly described as follows:

1. Six methods were used to identify the most informative cut-off points of our continuous variables to discriminate between the success and failure groups. Method 1 (ROC) is deduced from the ROC curve (Receiver Operating characteristic); the plot representing the rate of true positives to the rate of false positives. Method 2 (Md) reports a simple cut-off of the series at its median value. Method 3 (YI) uses the maximum value of the Youden index as the informative point [43]. Method 4 (ΔMinSnSp) refers to the minimum difference between the true-positive rate and the true-negative rate, allowing for maximum reduction of false diagnoses (false positives and false negatives). Method 5 (LRmax) is based on the positive likelihood ratio (LR+), which is the ratio of the true positive rate to the false positive rate. A high LR+ value would indicate how important it is in changing the probability generated by a positive diagnostic test. Method 6 (KLf/g or KLg/f) is based on the Kullback-Leibler probability distances (Dfǁg) and (Dgǁf) [44].
2. The use and crossing of these different methods allowed us to better probe our variables and select the most informative cut points. Joint investigations were also carried out to examine the selected cut points in clinical and physiological terms, and their concordance with the available literature, if any. The statistical and functional characteristics of the selected variables were also examined in light of the information provided by sensitivity, specificity, and likelihood ratios, as well as their discriminatory abilities expressed by the area under the ROC curve and the negotiation between false diagnosis rates.
3. For each dichotomous variable, a univariate analysis (chi-square) between the "success" and "failure" subgroups was performed to select variables with some predictive potential, using a liberal statistical criterion (P < 0.20). The corresponding positive likelihood ratio (LR+) was also considered so that a variable with LR+ ≥ 2 was also accepted, even if the Chi-square gave a P ≥ 0.20, which occurred in rare cases. In any case, only multivariate models that contained predictors that were all statistically significant were retained.
4. A three-step hierarchical modeling was performed. In each of these steps, a backward stepwise approach was used. The three steps are described as follows:
  - Step 1: consideration of class-A variables, which are the variables that should be considered for model fitting (confounding variables: age, sex, body mass index (BMI), with genetics driving age and sex differences [45], as well as variables that are specifically (theoretically) associated with this treatment, either in direct or indirect relation to lumbar stability;
  - Step 2: in addition to class-A variables, consideration of variables that may influence outcomes through adherence to the home exercise program [46, 47], here presumably considered as a mediator variable (class-B variables—potentially related to adherence);
  - Step 3: in addition to class-A and class-B variables, consideration of variables that may be associated with any exercise program (class-C variables—not treatment specific).
5. Various statistics to judge the performance of the selected multivariate models, including coefficients of determination (Nagelkerke’s R2), sensitivity (Se) and specificity (Sp) scores as well as positive and negative predictive values (PV+ and PV-) and likelihood ratios (LR+ and LR-) were calculated;

Class-A, -B, and -C variables are described in S1 File. Interpretation of the results at each step will help define the clinical significance of the CPR. Given that the purpose of the CPR is to predict whether or not a patient’s condition will improve with this exercise program, factors related to adherence to the home program (class-B variables), considered in step 2, are likely to be important. If these factors are problematic, they should be addressed before the exercise program is started. If class-C variables also have additional predictive value (Step 3), they should be concluded to be associated with any exercise program.

## Results

### Recruitment and attrition

**Fig 2:**
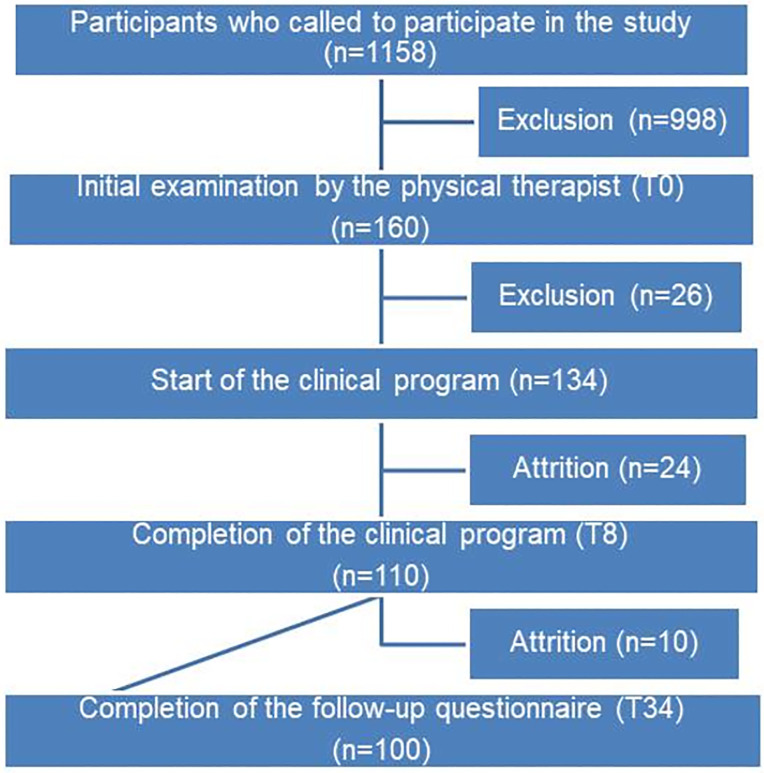
Flow diagram.

Participants were recruited mainly through newspaper advertisements, except six from the clinics as these usually start an exercise program before the end of the acute phase. The flow diagram (Fig 2) describes that from the initial 1158 calls, 160 participants were recruited for the first clinical evaluation, then 26 were excluded at the physical examination step. Of the 134 participants who started the exercise program, 24 participants (18%) left, most often due to factors deemed "non-preventable" (n = 16) by the research team (onset of pregnancy, relocation, surgery, death in the family, onset of neck pain, no response after the onset of COVID-19), but also for preventable reasons (7 for lack of attendance at appointments; 1 because of lack of patient-perceived improvement). At the six-month follow-up, an additional attrition of 10 participants was observed, most of them not giving any reason (no response to our e-mails).

### Characteristics of the patient sample and change in clinical outcomes during the LSEP

Participants’ demographic, anthropometric and clinical characteristics at baseline (T0) were equivalent between males (n = 50) and females (n = 60), with the exception of height and weight, as expected (Table 1). Overall, these participants had moderate disability and pain. Among the ethnic groups considered, these 110 participants were distributed as follows: American Indian/Alaska Native (n = 0), Asian (n = 4), Black/African American (n = 11), Hispanic/Latino (n = 6), Hawaii native/Pacific Islander (n = 0), White (n = 81) and others (n = 8).

**Table 1:** Demographic, anthropometric and clinical characteristics of the participants.

Variables	Men (n = 50)		Women (n = 60)		T-test
	Mean	(SD)	Mean	(SD)	P value
Age (yrs)	44	(11)	43	(13)	0.640
Height (m)	1.75	(0.07)	1.64	(0.06)	**< 0.001**
Mass (kg)	82	(16)	71	(13)	**< 0.001**
BMI (kg/m^2^)	26.6	(5.0)	26.4	(4.5)	0.758
ODI (%)	25.6	(9.0)	27.8	(10.6)	0.251
NPRS (score /10)	5.2	(1.5)	5.0	(1.2)	0.450
PCS (score /52)	22.9	(12.0)	18.3	(11.7)	**0.045**
IDPtot (score/100)	23.7	(15.0)	24.3	(13.4)	0.824
StarTBack (score/9)	4.4	(2.0)	3.7	(2.0)	0.104
Duration LBP*	/	/	/	/	/

BMI: Body mass index; ODI: Oswestry disability index; NPRS: Numerical pain rating score; PCS: Pain catastrophizing scale; PDItot: Psychological distress inventory (total score); StarT Back: screening tool.

Most participants had psychological characteristics below the thresholds of clinical significance according to PCS (threshold: 30/52), or near or on these thresholds as revealed with the PDI (threshold: 26%), and the StarT Back screening tool (threshold: 4/9).

*For the duration of the self-reported LBP, 97% (107/110) of participants had chronic pain (3 months or more), distributed as follows [48]: less than one month (n = 0), 1–3 months (n = 2), 3–6 months (n = 2), 6–12 months (n = 12), (5) 1–5 years (n = 40), (6) >5 years (n = 54).

All the participants completed the 8-week exercise program and reached the third (final) phase of the program. Home-exercise adherence was high at T8 (ratio: 0.85 ± 0.21) and lower at T34 (ratio: 0.51 ± 0.40). The participants meeting the criteria for success, clinically significant improvement and failure at T8 were 54 (23 males + 31 females), 11 (4 M + 7 F) and 45 (23 M + 22 F), respectively. At T34, they were 53 (25 M + 28 F), 11 (1 M + 10 F) and 36 (16 M + 20 F), respectively. Overall, the participants assessed at T8 (n = 110) showed a significant decrease (pre- to post-treatment) in the ODI and NPRS (Table 2) resulting in strong effect sizes (Cohen’s *d* = -1.24 and -1.70, respectively). Significant decreases of these outcomes were also observed at T34 (n = 100), also resulting in strong effect sizes (Cohen’s *d* = -1.24 and -1.32, respectively).

**Table 2:** Primary Outcome Measures (OUTs) for the 110 participants who completed the 8-week exercise program (A) as well as for the 100 participants who completed the six-month follow-up (B), also ranked by level of treatment success.

A. Data for participants at T8	Time	All (n = 110)		Failure (n = 45)		Improvement (n = 11)		Success (n = 54)	
ODI (%)*	T0	27	(10)	24	(9)	36	(9)	27	(10)
	T8	14	(11)	22	(10)	21	(7)	7	(5)
NPRS (/10)	T0	5.0	(1.3)	5.3	(1.4)	5.2	(1.6)	4.8	(1.2)
	T8	2.7	(1.4)	3.4	(1.4)	3.0	(1.2)	2.1	(1.0)
B. Data for participants at T34	Time	All (n = 100)		Failure (n = 36)		Improvement (n = 11)		Success (n = 53)	
ODI (%)*	T0	27	(10)	23	(8)	37	(9)	27	(9)
	T34	14	(11)	23	(10)	24	(10)	6	(4)
NPRS (/10)	T0	5.0	(1.3)	5.1	(1.5)	5.7	(1.1)	4.9	(1.2)
	T34	3.0	(1.7)	3.9	(1.8)	3.8	(1.9)	2.1	(1.1)

* Variable used to determine membership in the "Failure", "Improvement", and "Success" subgroups.

### Derivation of CPRs of success

Candidate variables to multivariate logistic models are presented in S2 File, with details regarding between-group comparisons (Chi-square test) as well as their indicators of diagnosis performance (Se, Sp, LR+, LR-).

As detailed in S3 File, different multivariate logistic models were developed as candidates for the CPR for success at T8 (n = 9 models) and at T34 (n = 16 models), showing which class-A, class-B and class-C variables were retained as the models built-up. Also provided in S3 File is a rationale to choose the best model for each assessment time (T8 and T34), considering the different indicators of diagnosis performance as well as their usability for the therapist and patient.

#### CPR for success at the end of the treatment (T8)

**Fig 3:**
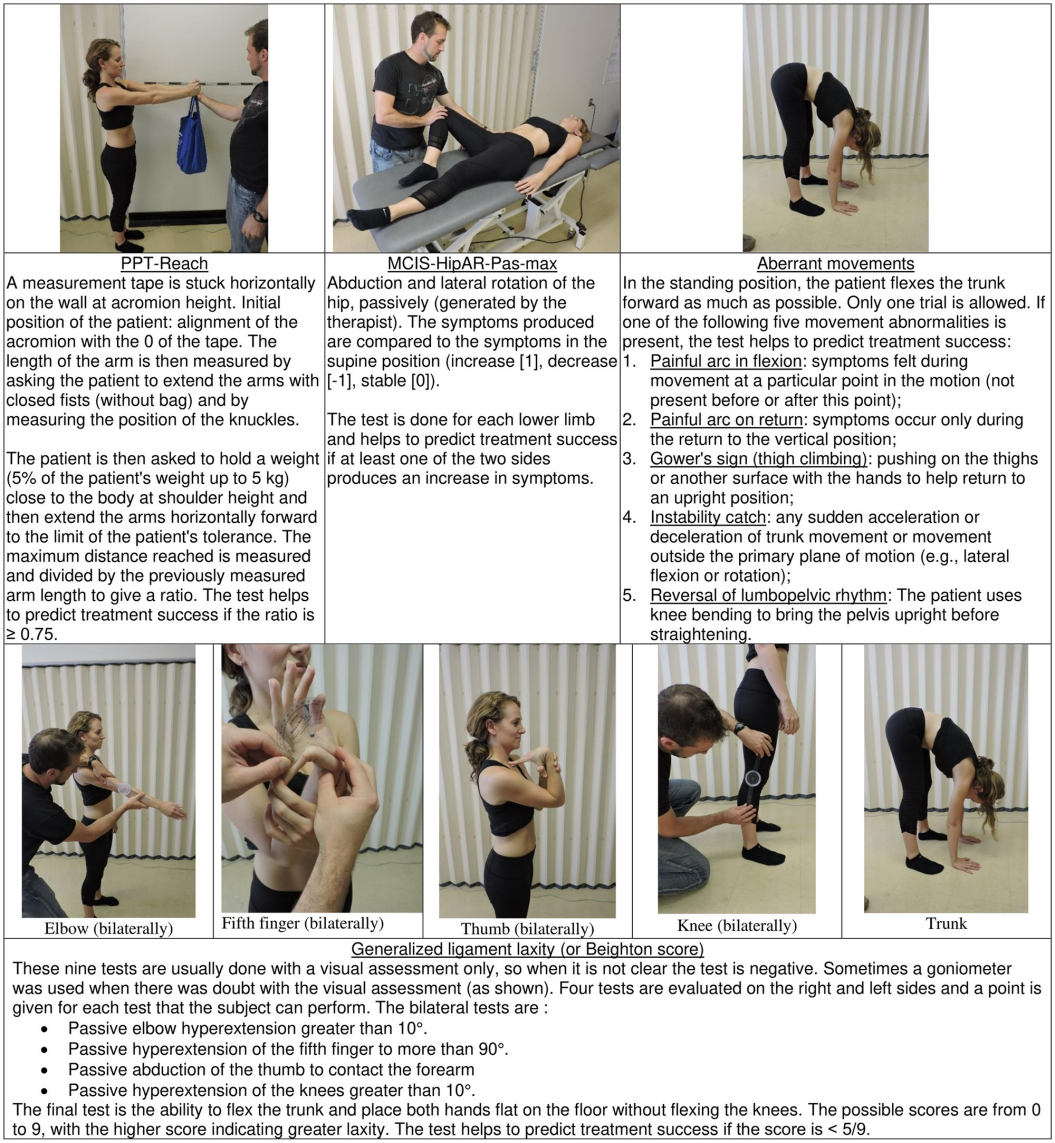
Illustration and brief description (more detailed in S1 File) of the four clinical tests retained in the CPR of success at the end of treatment (at time T8).

The selected CPR for success at T8 (model 3; details in Table 3, part A) comprises only the following four class-A variables: 1. a loaded reach physical performance test (PPT-Reach) ≥ 0.75/1, 2. a positive motor control impairment test involving a passive hip abduction and rotation (MCIS-HipAR-Pas-max), 3. a positive aberrant movement test (Abe-Mvt) and 4. a general laxity score (Beighton score) ˂ 5/9. These four tests are illustrated and described in Fig 3. The corresponding indicators of diagnosis performance are also detailed in Table 3, part A.

**Table 3:** Coefficients, indicators of diagnostic performance as well as explanatory and adjustment indicators of the selected predictive models for the CPR at T8 (A) and T34 (B).

**A. CPR for success at T8**		**B. CPR for success at T34**	
Selected variables	B (CI 95%)	Selected variables	B (CI 95%)
PPT-Reach ≥ 0,75/1	1,78 (0,32–3,23) ^3^	MCIP-HipE-Act-max negative	1,13 (0,11–2,15) ^1^
MCIS-HipAR-Pas-max positive	1,58 (0,59–2,57) ^3^	TME-abdominals ≥ 72,62 s	1,31 (0,14–2,48) ^2^
Abe-Mvt positive	1,40 (0,30–2,50) ^2^	PPT-Reach ≥ 0,76/1	2,07 (0,45–3,69) ^3^
Beighton ˂ 5/9	1,65 (0,00–3,30) ^1^		
Indicators of diagnosis performance		Indicators of diagnosis performance	
Sensitivity	35,2 (22,4–47,9)	Sensitivity	22,64 (11,37–33,9)
Specificity	95,6 (89,5–100)	Specificity	97,22 (91,85–100)
Positive likelihood ratio (LR+)	**7,91** (1,95–32,2)	Positive likelihood ratio (LR+)	**8,15** (1,11–59,9)
Negative likelihood ratio (LR-)	0,68 (0,55–0,83)	Negative likelihood ratio (LR-)	0,80 (0,68–0,93)
Positive predictive value (PV+)	**90,5** (77,9–100)	Positive predictive value (PV+)	**92,3** (77,8–100)
Negative predictive value (PV-)	55,1 (44,1–66,2)	Negative predictive value (PV-)	46,1 (34,8–57,3)
Area under the curve(AUC), P value*	0,78 (0,68–0,87)	Area under the curve(AUC), P value*	0,73 (0,62–0,83)
Post-treatment probability of success (%) †	88 (68–96)	Post-treatment probability of success (%) †	90 (61–98)
Explanatory and adjustment indicators		Explanatory and adjustment indicators	
Nagelkerke’s R^2^ coefficient (%)	32,2	Nagelkerke’s R^2^ coefficient (%)	23,4
Hosmer & Lemeshow Test (χ2, p)	(χ^2^ = 4,31; *P* = 0,37)	Hosmer & Lemeshow Test (χ2, p)	(χ^2^ =, 11; *P* = 0,99)

B: Beta Coefficient (Log odds); ()^n^ Ranking of predictors according to the Adequacy statistic (n = 1 means that it represents the most influential predictor; the same rank can be assigned when this indicator gives the same score). The Adequacy statistic gives the individual explanatory value of the predictor and thus its explanatory strength. It is the ratio of -2log-likelihood (-2LL) of the predictor to -2LL of the complete Model, and represents the proportion of the log-likelihood of the complete Model (considering all the predictors) that is explained by each predictor individually.

* P values are all ˂ 0.001; Hosmer & Lemeshow Test: goodness-of-fit test (must be nonsignificant).

^†^ Estimated with Fagan nomogram (http://araw.mede.uic.edu/cgi-bin/testcalc.pl), knowing LR+, LR-, and estimating prevalence (at T8: 54/110 patients, or 49%; at T34: 53/100 patients, or 53%). For CPR for success at T8, this indicates that the probability of success increases from 49% (without CPR use) to 88% (with CPR use).

The number of participants in the success and failure groups was determined based on the number of positive tests of the CPR (Table 4, part A). Using these numbers, the corresponding performance statistics were also generated (Table 5, part A). From the information in these two tables, it can be concluded that the best combination for predicting treatment success appears when two or more predictors (or clinical tests) are positive (or achieved) because this condition generates a clinically greater LR+ of 17.91 as well as the highest probability of success (95.6%).

**Table 4:** Number of participants in the success and failure groups as a function of the number of positive tests of the CPR for success at T8 (A) and T34 (B).

**A. CPR for success at T8**		
Number of positive tests	Successful outcome group (n = 54)	Failure outcome group (n = 45)
1 or more	54	40
2 or more	43	15
3 or more	10	0
4	1	0
**B. CPR for success at T34**		
Number of positive tests	Successful outcome group (n = 53)	Failure outcome group (n = 36)
1 or more	41	15
2 or more	12	1
3	2	0

**Table 5:** Performance statistics (95% confidence intervals) associated with the numbers of predictors present in the CPR for success at T8 (A) and T34 (B).

Number of positive tests	Sensitivity	Specificity	Positive likelihood ratio (LR+)	Probability of success (%) or PV+ after the LSEP
**A. CPR for success at T8**				
1 or more	18,51 (8,2–28,9)	97,8 (93,5–100)	8,33 (1,11–62,6)	90,9 (73,9–100)
2 or more	79,6 (68,9–90,4)	95,5 (89,5–100)	17,91 (4,59–69,9)	95,6 (89,5–100)
3 or more*	100 (100–100)	93,3 (80,7–100)	15,0 (2,3–99,6)	91,0 (73,4–100)
4	-	-	-	-
**B. CPR for success at T34**				
1 or more	29,3 (17,02–41,52)	93,33 (85,15–100)	4,39 (1,21–15,98)	87,0 (70,83–100)
2 or more *	22,6 (11,37–33,9)	97,25 (91,8–100)	8,15 (1,11–59,9)	92,3 (77,8–100)
3	-	-	-	-

LSEP: lumbar stabilization exercise program. PV+: positive predictive value

* With three or more predictors, the headcounts are significantly reduced (at T8: 10 successes and 0 failure; at T34: 12 successes and 1 failure), as described in Table 3, making the calculation of statistical indicators impossible (failure group = 0 or 1). As done in other studies [49, 50], an approximation of the indicators was therefore estimated, for illustrative purposes, using the numbers in the failure group corresponding to the condition with two or more predictors (n = 15).

#### CPR for success at the six-month follow-up (T34)

**Fig 4:**
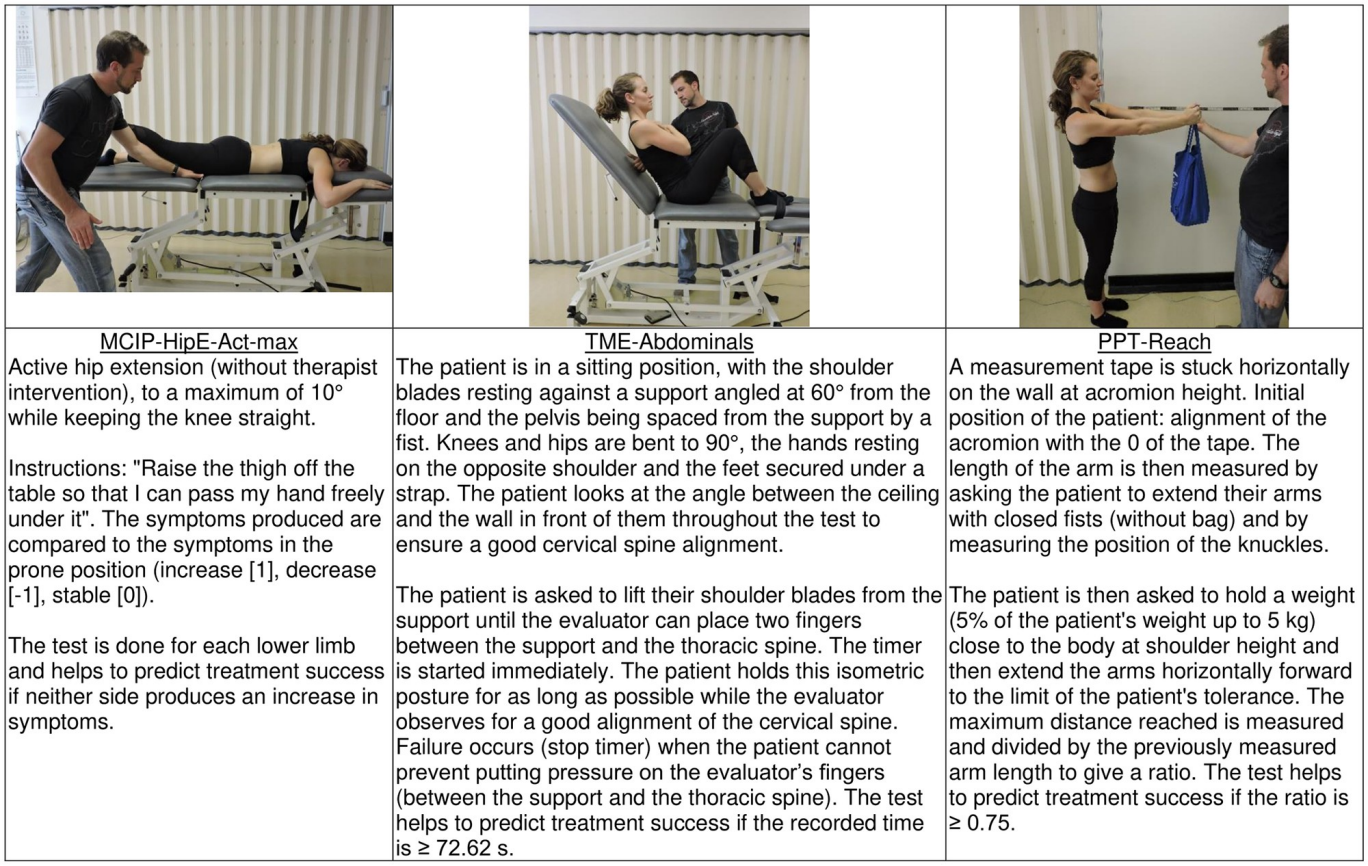
Illustration and brief description (more detailed in S1 File) of the three clinical tests retained in the CPR of success at the six-month follow-up (at time T34).

The selected CPR for success at T34 (also model 3; details in Table 3, part B) comprises only the following three class-A variables: 1. a loaded reach physical performance test (PPT-Reach) ≥ 0,76/1, 2. a negative motor control impairment test involving an active hip extension (MCIP-HipE-Act-max) and 3. an abdominal muscles endurance test (TME-Abdominals) ≥ 72.62 s. The three tests are illustrated and described in Fig 4. The corresponding indicators of diagnosis performance are also detailed in Table 3, part B.

The number of participants in the success and failure groups was determined based on the number of positive tests of the CPR (Table 4, part B), as well the corresponding performance statistics (Table 5, part B). The best combination for predicting treatment success appears when two or more predictors (or clinical tests) are positive (or achieved) because this condition generates a clinically greater LR+ of 8.15 as well as the highest probability of success (92.3%).

## Discussion

Our data and analysis allowed for the derivation of two CPRs of success for an LSEP, one at the end of the program (done once before [12]) and one at six-month follow-up. The corresponding indicators of diagnostic performance were good and the usability was deemed acceptable as only three or four clinical tests were needed for the CPRs. Moreover, the tests included in the CPRs measure concepts (class-A variables) with a clear relationship to spinal instability, providing a specific link to the treatment provided (LSEP).

### Predictive value of the selected CPRs

The selected CPR for success at each assessment time (T8 and T34) included only class-A variables (Table 3), which allows for comparison of their overall performance. In both cases, when two or more tests are positive, a much higher performance is estimated at T8 (LR+ = 17.9) than at T34 (LR+ = 8.2). These LR+ scores, as well as the corresponding probabilities of success at the end of treatment (96% and 92% respectively), are comparable or better than various CPRs developed for physiotherapy treatments related to the spine (Table 6). They are also higher than the preliminary CPR for success (at T8) derived earlier [12] for lumbar stabilization exercises (LR+ = 4.0; probability of success = 67%).

**Table 6:** Comparison of the performance of different CPRs of success developed for different treatments offered in physiotherapy.

CPR for success study	Physical therapy treatment	LR+	Probability of success without/with the CPR (difference)
Present study–at T8	Lumbar stabilization exercises	**17,9**	49 / **96%** (Δ = +47%)
Present study–at T34	Lumbar stabilization exercises	**8,2**	53 / **92%** (Δ = +39%)
Hicks, Fritz [12]	Lumbar stabilization exercises	4,0	33 / 67% (Δ = +34%)
Stolze, Allison [49]	Pilates exercises	10,6	54 / 93% (Δ = +39%)
Cai, Pua [51]	Spinal traction	9,4	19 / 69% (Δ = +50%)
Cleland, Childs [52]	Thoracic spine manipulation	5,5	54 / 86% (Δ = +32%)
Flynn, Fritz [50]	Spinal manipulation	24,4	45 / 95% (Δ = +50%)

One interpretation of positive likelihood ratios for evidence-based healthcare [53] suggest that: 1. LR+ > 10 generate large and often conclusive changes between pretreatment to posttreatment probability; 2. LR+ of 5 to 10 generate moderate changes between pretreatment and posttreatment probability; 3. LR+ of 2 to 5 generate small (but sometimes large) changes in probability; 4. LR+ of 1 to 2 change probability to a small (and rarely large) degree. Our CPR at T8 (LR+ = 17.9) fits into the first category (predicts large and often conclusive changes), while the CPR at T34 (LR+ = 8.2) fits the second (predicts moderate changes).

### Theoretical justification of the predictors selected in the CPRs

The CPRs of success at T8 and T34 comprised solely class-A variables. Their theoretical justification is discussed here. The reader interested in the possible role played by class-B and class-C variables in the prediction of the LSEP success (corresponding to other multiple regression models) can find a discussion in S4 File.

#### PPT-Reach physical performance test

PPT-Reach was selected in the final CPRs, at both T8 and T34, with almost identical cutoffs (T8: ≥0.75/1; T34: ≥0.76/1). This test involves standing with a weighted bar (5% of body weight, up to 4.5 kg) held at shoulder height and then extending the arms, parallel to the ground, are far as possible while maintaining an upright posture. The ratio represents the distance reached vs. arm length. This test assesses the role of the stabilizing muscles of the lumbar spine in a neutral posture, in which passive tissue stiffness plays a minimal role [54]. Raising the load above the body’s center of mass (shoulder height) increases the challenge to spine stability (by increasing the potential energy of the system), as evidenced by increased trunk muscle activation with higher loads [55, 56]. This is further challenged when the supported load is held away from the body, as not only is dorsal trunk muscle activation increased to counteract the net moment in flexion (also producing added spine compression), but there is also an increase in abdominal muscle activation that can only be attributed to maintaining lumbar stability [55, 57]. Our original hypothesis was that patients with poorer lumbar spine stabilization would have difficulty performing this test (scores below 0.75/1) and would benefit from the lumbar stabilization exercise program. However, it is the patients with higher scores who benefited. Moving the load forward induces an automatic co-contraction of the trunk muscles [57], which would contribute to maintaining lumbar stability [55]. This test, therefore, may not elicit pain related to lumbar instability, but might elicit pain from other causes such as spinal compression. Patients who would benefit from an LSEP could learn to use similar co-contraction strategies during different tasks. Patients who have pain that is not controlled with trunk muscle co-contraction, on the other hand, would not benefit from training these motor control strategies.

The same rationale underlies the inclusion of the prone instability test (ProneIT variable) in a previous derivation of a CPR for LSEP success [12]. While the PPR-Reach requires some equipment (bag with weights and measuring tape), it is a ratio-scale measure (in cm) with almost perfect inter-rater reliability (intraclass correlation or ICC: 0.99) and excellent between-day reliability (ICC: 0.91) [58], making it ideal for inclusion in a CPR. The ProneIT, on the other hand, has lower reported reliability (see S1 File), possibly related to the fact that its interpretation is more subjective and it requires manual intervention by the therapist.

#### MCIP-HipE-Act-max variable

The MCIP-HipE-Act-max variable (final CPR at T34) is derived from an MCI test and requires active unilateral hip extension while lying prone (until the thigh no longer touches the examination table). Limb movements, especially when only one limb is involved, usually cause asymmetric forces and moments at the lumbar level. It has been proposed that an inability to limit lumbar movement during limb movement would be indicative of lumbar instability, thus causing symptoms [59]. Our data, however, indicate that the MCIP-HipE-Act-max test must be negative to predict success with LSEP (active hip extension in prone causes no change, or a reduction, in symptoms). This result appears to be consistent with the same pattern of higher scores on the PPT-Reach and better trunk muscle endurance (TME-Back and TME-Abdominals; see below) being predictive factors for success. Patients who already have better trunk muscle function (on at least some measures) may see faster benefits from the stabilization exercise program (i.e., need only 8 weeks of training to see a functional benefit) and/or may be more likely to retain these functional benefits longer once they stop training (i.e., at the six-month follow-up).

#### MCIS-HipAR-Pas-max variable

The MCIS-HipAR-Pas-max variable (final CPR at T8) is derived from an MCI test in which the therapist passively moves the hip from a unilateral crook-lying position into abduction and external rotation. While the association between this variable and lumbar instability is not obvious, because the test is passive, a positive finding may indicate a relatively lower passive rotational stiffness in the lumbar spine, compared to the hip, inducing pain in the lumbar spine as it is pulled into rotation with the movement of the lower limb. Learning to actively stabilize (stiffen) the lumbar spine, with an LSEP, may benefit patients who experience pain with this test, allowing then to control and limit spine rotation during movements at the hip. It should be noted, however, that this pain provocation test may not be specific to patients who require an LSEP to eliminate these symptoms. It also cannot be ruled out that this test caused irritation of the sacroiliac joint (SI joint dysfunction was not an exclusion criterion), which is under tension during this test. Whether or not this test is specific to a lumbar instability disorder will be determined at the validation stage of the CPR.

#### Aberrant movements (Abe-Mvt)

The presence of aberrant movements (Abe-Mvt; final CPR at T8) was also retained in the CPR of Hicks, Fritz [12], as well as in a subsequent attempt to validate this CPR [15] in which only Abe-Mvt and ProneIT were retained. Aberrant movements are associated with decreased control of lumbar segment mobility in the central (i.e., neither beginning nor end) portion of the lumbar ROM, as demonstrated by a fluoroscopic study of flexion and extension movements in the sagittal plane [14]. Further studies on aberrant movements support the construct validity of this test, but also note that the method of operationalizing this test does not distinguish subgroups of patients with different pathologies [60].

#### Generalized ligament laxity

Joint hypermobility syndrome, as diagnosed with the Beighton scale, is thought to be caused by a decrease in collagen content in tendons, ligaments, joint capsules, and skin, which in turn reduces their stiffness and thus may have a relationship with joint instability and pain [61]. A relationship with sex is expected [62], and was observed in our sample with a higher score (Wilcoxon test; P ˂ 0.001) in females (2.2 ± 2.6; n = 53) than in males 0.5 ± 1.4 (0.5 ± 1.4; n = 46). A systematic review concluded that these individuals also have poorer proprioception [63], which has the potential to increase joint instability. It was expected, therefore, that patients with a high (not low) Beighton scale score would be more likely to demonstrate lumbar instability, due to low passive spine stiffness, and to benefit from the LSEP. The opposite was found.

A low score for generalized ligament laxity (Beighton variable ˂ 5/9) was retained in the final CPR at T8. One speculative explanation is that individuals with a high Beighton score, presumably from birth, would have already learned to actively control the segmental movements of the spine, and would not derive added benefit from an 8-week LSEP. Another is that generalized tissue laxity would make a transient loss of inter-segmental motor control, leading to movement beyond a normal physiological range (transient segmental buckling [64]), less likely to cause injurious or painful loading of surrounding tissues. In other words, these individuals may have larger margin for error for segmental motor control in the spine and are therefore less likely to experience LBP associated with lumbar instability and/or benefit from an LSEP.

#### Back and abdominal muscle endurance (variable TME-Back and TME-Abdominals)

Back muscle endurance (variable TME-Back > 225 s) was not retained in the final CPR at T8 (but was present in the other models; S3 File) but abdominal muscle endurance (variable TME-Abdominals > 72.62 s) was retained in the final CPR at T34. Interestingly, model 6 at T8 (not shown) excluded TME-Back and replaced it with TME-Abdominals and BMI, but these two variables just failed to reach statistical significance (P = 0.064 and 0.065, respectively). Such results were not observed by Hicks, Fritz [12], possibly because of their study included patients with a shorter symptom duration than in the present study. Corresponding results have, however, been found in other studies. Davarian et al. [65] found that patients with chronic LBP who responded positively to the Hicks, Fritz [12] CPR for success demonstrated greater back muscle endurance during a relative-effort-based (60% of maximal strength) endurance test. Positive correlations have also been reported between a back muscle endurance test (prone bridge test) and various lumbar instability tests performed in the present study (Mvt-Abe, Instability-Man, Instability-Ext, EJT-Act-Doul) [66].

Trunk muscle endurance is relevant because of the protective effect that it is thought to play on lumbar stability, as promoted by McGill [4]]. Poor back muscle endurance predicts both a first episode of LBP [67] and prolonged disability [68]. Trunk muscle endurance is also negatively correlated with symptom duration [66], possibly explained by a gradual transformation of muscle fibers into more "fatigable" fiber types in chronic LBP [69]. Better trunk muscle endurance as a predictor of success with an 8-week LSEP could mean that patients with poor endurance require more than eight weeks of training to reverse the underlying causes of their LBP. Those who respond, on the other hand, may have lumbar instability related to deep muscle inhibition (LuM, TrA), and may even overuse the superficial muscles [70]. These patients might still perform well on tests of trunk muscle endurance, but benefit from specific motor control training. They may also maintain those benefits more effectively because they already have good muscle endurance in the larger superficial muscles.

An alternative explanation would be that TME variables are simply prognostic factors, indicating which patients are most likely to recover regardless of the underlying cause of the LBP. This would still justify the use of TME exercises for patients with LBP but would make the inclusion of TME variables in a CPR unnecessary. For the different CPR models considered in our data analysis, including TME-Back in model 5 produced only a minor improvement over model 3, which did not include this measure but was otherwise identical (**S3-1 & S3-2 Tables in** S3 File). This could justify the elimination of this test at the initial clinical exam, as this considerably reduces the assessment burden for both the patient and therapist. On the other hand, measures of trunk muscle endurance (TME-Back, TME-Abdominals, TME-Side) would still be useful as objective measures of patient progress, during and after an LSEP, which could justify their clinical use.

### Which CPR to choose? To predict success at the end of treatment (T8) or at the six-month follow-up (T34)?

To the authors’ knowledge, this is the derivation of CPRs to predict LSEP success both immediately after treatment and at six-month follow-up. Ideally, both CPRs (at T8 and T34) would now proceed to stages 2 (validation) and 3 (impact study) of development [71]. However, because these development stages are so resource intensive, it is much more realistic to select only one CPR for further development.

Most arguments support the choice of the CPR at T8. In terms of diagnostic performance indicators, when at least two variables are present, the CPR at T8 offers a better performance (LR+ = 17.91; VP+ = 95.6) than the CPR at T34 (LR+ = 8.15; VP+ = 92.3). In terms of usability by therapists, the CPR at T34 is also more burdensome, despite the CPR at T8 having four variables and the CPR at T34 having only three. This is because of the inclusion of a time consuming, and fatiguing, abdominal muscle endurance test (TME-Abdominals) for the CPR at T34.

In the 100 patients who completed the six-month follow-up, the effects of the LSEP were both positive at T8 and well maintained at T34, both for perceptions of disability (ODI: effect sizes of -1.20 and -1.30 at T8 and T34, respectively) and for pain intensity (effect sizes of -1.70 and -1.35 at T8 and T34, respectively). These results are in agreement with a meta-analysis [72] indicating that an LSEP maintains its post-treatment effects (disability, pain) better than any other comparator (passive, inactive treatments or other types of exercise). It is therefore reasonable to expect that the use of a CPR at T8 has some predictive value for outcomes at T34.

## Conclusion

Two CPRs of success for an LSEP were derived, one at the end of the program and one at six-month follow-up. Their diagnostic performance was good and usability acceptable as only three or four clinical tests were needed for the CPRs. Finally, the tests included in the CPRs measure concepts that have a clear relationship to spinal instability, providing a specific link to the treatment provided. These results support the further development of these CPRs by proceeding to the validation stage.

## Supporting information

S1 File Description of all measures. Description of tools or procedures used to perform all measures, their corresponding interrater reliability, their variable abbreviations and whether they were considered as class A, B or C candidate predictors.
(DOCX)

S2 File Diagnosis performance indicators for individual variables. Pearson’s Chi-square P values, sensitivity, specificity as well as positive and negative likelihood ratios (LR+ and LR-) corresponding to each candidate predictor.
(DOCX)

S3 File Logistic models and selection of final CPRs. Logistic regression multivariate models for success at the end of treatment (T8) and at the six-month follow-up (T34) and rational to select the final clinical prediction rule of success (best models) at each time point.
(DOCX)

S4 File Discussion about class-B and class-C variables. Rationale for the inclusion of class-B and class-C variables in the multivariate logistic models for success following a lumbar stabilization exercise program.
(DOCX)
