# Supplementary material for: Derivation of clinical prediction rules for identifying patients with non-acute low back pain who respond best to a lumbar stabilization exercise program at post-treatment and six-month follow-up
Source: PLoS One. 2022 Apr 27;17(4):e0265970. doi: 10.1371/journal.pone.0265970 (PMC9045609; doi:10.1371/journal.pone.0265970)
Supplement: S2 File — Pearson’s Chi-square P values, sensitivity, specificity as well as positive and negative likelihood ratios (LR+ and LR-) corresponding to each candidate predictor. (DOCX) [file pone.0265970.s002.docx]

**SUPPORTING FILE 2**

Only variables that showed a tendency (P < 0.20) to discriminate between success and failure groups (Chi-square test) or had a LR+ > 2 were retained as candidates for multivariate analyses and are presented in this file.

Candidate variables for the success CPR at T8 are presented in Table S2-1 for variables initially continuous in nature and in Table S2-2 for variables initially dichotomous in nature. A total of 22 class-A variables, 9 class-B variables, and 10 class-C variables were candidates to proceed to the multivariate analysis stage.

Candidate variables for the success CPR at T34 are presented in Tables S2-3 (initially continuous variables) and S2-4 (initially dichotomous variables), for a total of 17, 8, and 7 variables for class-A, class-B, and class-C, respectively.

Variable acronyms are explained in S1 file.

**Table S2-1. Comparisons between success (n = 54) and failure (n = 45) subgroups at time T0 for continuous variables (which were dichotomized) - Results for participants used for CPR at T8.**

| Variables*, cut-off | Pearson’s Chi-square | Indicators of diagnosis performance | | | |
| --- | --- | --- | --- | --- | --- |
| (dichotomization method†) | *P* value ‡ | Se | Sp | LR+ | LR- |
| Class-A variables |  |  |  |  |  |
| **Beighton ˂ 5/9** (KL) | 0,115 | 93 | 18 | 1,13 | 0,42 |
| Lordosis ˂ 24,5° (IY) | **0,034** | 37 | 82 | 2,08 | 0,77 |
| Lordosis ˂ 29° (∆MinSnSp) | 0,091 | 59 | 58 | 1,40 | 0,71 |
| BMI ≥ 29,37 kg/m^2^ (ROC) | 0,080 | 28 | 87 | 2,08 | 0,83 |
| BMI ≥ 33,3 kg/m^2^ (LRmax) | **0,037**‡ | 15 | 98 | 6,67 | 0,87 |
| TME-Abdominals ≥ 71,9 s (ROC) | **0,043** | 33 | 84 | 2,14 | 0,79 |
| **TME-Back ≥ 225,56 s** (LRmax) | 0,088 | 15 | 96 | 3,33 | 0,89 |
| PPT-SitStand ≥ 7,6 s (LRmax) | **0,044**‡ | 98 | 13 | 1,13 | 0,14 |
| PPT-Flexion ≥ 7,48 s (ROC) | 0,050 | 70 | 49 | 1,38 | 0,61 |
| PPT-Flexion ≥ 5,97 s (KLg/f) | 0,075‡ | 96 | 16 | 1,14 | 0,24 |
| **PPT-Reach ≥ 0,75/1** (ROC) | **0,007** | 28 | 93 | 4,17 | 0,77 |
| PPT-Reach ≥ 0,8/1 (LRmax) | 0,068‡ | 13 | 98 | 5,83 | 0,89 |
| Class-B variables |  |  |  |  |  |
| Treatment expectation ˂ 3,72 /5 (KLg/f) | 0,177 | 24 | 87 | 1,81 | 0,88 |
| **FABQ-PA ≥ 6,5/24** (LRmax) | 0.199 | 93 | 16 | 1,10 | 0,48 |
| HPA-leisure≥ 1,62 /5 (ROC) | 0,111 | 98 | 9 | 1,08 | 0,21 |
| Illness perception ≤ 44 /80 (ROC) | 0,052 | 57 | 62 | 1,52 | 0,68 |
| **Illness perception** **≤ 38,5 /80** (KLf/g) | **0,033** | 31 | 87 | 2,36 | 0,79 |
| PDIang ˂ 54,17 /100 (KLg/f) | 0,075‡ | 96 | 16 | 1,14 | 0,24 |
| PDIdep ˂ 2,8 /100 (KLg/f) | 0,080 | 33 | 82 | 1,88 | 0,81 |
| SSES-FamPa ˂ 34,5 /100 (KLf/g) | **0,019** | 93 | 24 | 1,23 | 0,30 |
| Class-C variables |  |  |  |  |  |
| PelvisFlx-ROM ˂ 73° (KLg/f) | 0,095 | 50 | 67 | 1,50 | 0,75 |
| LumbLatFlx-ROM-Min ˂ 19° (KLg/f) | **0,005** | 44 | 82 | 2,50 | 0,68 |
| LumbFlx-ROM ≥ -3° (ROC) | 0,051 | 20 | 93 | 3,06 | 0,85 |
| MCIP-HipER-Pas-ROM-min ˂ 61° (KLf/g) | **0,008** | 67 | 60 | 1,67 | 0,56 |
| MCIP-HipIR-Pas-ROM-min ˂ 51° (KLf/g) | **0,022**‡ | 98 | 16 | 1,16 | 0,12 |
| PSLR-Pain ROM-min ˂ 67° (KLf/g) | 0,090 | 83 | 31 | 1,21 | 0,54 |
| PSLR-Max ROM-Moy ˂ 92,5° (KLf/g) | 0,135 | 89 | 22 | 1,14 | 0,50 |
| PSLR-Max ROM-Min ˂ 51° (KLf/g) | 0,106‡ | 15 | 96 | 3,33 | 0,89 |
| PSLR-Max ROM-Min ˂ 97° (KLg/f) | 0,089‡ | 98 | 11 | 1,10 | 0,17 |
| NPRS ≤ 6,5 / 10 (KLg/f) | **0,014** | 94 | 22 | 1,21 | 0,25 |

* Only variables that showed a trend (P < 0.20) with Pearson's Chi-square test or had an LR+ > 2 were retained as candidates and presented in this table, with the exception of the model fit variables (age, BMI). Variables retained in the multivariate logistic models are in bold type.

† Dichotomization method: IY: Youden index; KL: Kullback-Leibler distances; LRmax = maximization of likelihood ratio (LR); Med: median; ROC = ROC curve optimum point; ∆MinSnSp: minimum difference between the rate of true positives and the rate of true negatives.

‡ Fisher's exact test for small numbers. P values ≤ 0.05 are in bold type.

**Table S2-2. Comparisons between success (n = 54) and failure (n = 45) subgroups at time T0 on dichotomous variables - Results for participants used for CPR at T8.**

| Variables* | Pearson’s Chi-square | Indicators of diagnosis performance | | | |
| --- | --- | --- | --- | --- | --- |
|  | *P* value ‡ | Se | Sp | LR+ | LR- |
| Class-A variables |  |  |  |  |  |
| Sex (female) | 0,397 | 57 | 51 | 1,17 | 0,83 |
| ASLR-Act-Pain-max positive | **0,013** | 46 | 78 | 2,08 | 0,69 |
| ProneIT negative | **0,042** | 65 | 56 | 1,46 | 0,63 |
| **Abe-Mvt positive** | **0,022** | 39 | 82 | 2,19 | 0,74 |
| MCIP-HipER-Act-max positive | 0,191 | 46 | 67 | 1,39 | 0,81 |
| MCI4-ShoF-Act-max positive | 0,138 | 33 | 80 | 1,67 | 0,83 |
| MCIT-KneeE-Pas-max positive | 0,095 | 50 | 67 | 1,50 | 0,75 |
| MCIT-KneeE-Act-max positive | 0,136 | 48 | 67 | 1,44 | 0,78 |
| **MCIS-HipAR-Pas-max positive** | **0,004** | 48 | 80 | 2,41 | 0,65 |
| MCIS-HipAR-Act-max positive | **0,042** | 39 | 80 | 1,94 | 0,76 |
| Class-B variables |  |  |  |  |  |
| StageChange-D2 positive | **0,042**‡ | 100 | 9 | 1,10 | 0,00 |
| Class-C variables |  |  |  |  |  |
| None |  |  |  |  |  |

* Only variables that showed a trend (P < 0.20) with Pearson's Chi-square test or had an LR+ > 2 were retained as candidates and presented in this table, with the exception of the model fit variable (Sex). Variables retained in the multivariate logistic models are in bold type.

‡ Fisher's exact test for small numbers. P values ≤ 0.05 are in bold type.

**Table S2-3. Comparisons between success (n = 53) and failure (n = 36) subgroups at time T0 for continuous variables (which were dichotomized) - Results for participants used for CPR at T34.**

| Variables*, cut-off | Pearson’s Chi-square | Indicators of diagnosis performance  (‡‡) | | | | | | | |
| --- | --- | --- | --- | --- | --- | --- | --- | --- | --- |
| (dichotomization method†) | *P* value ‡ | Se | | Sp | | LR+ | | LR- | |
| Class-A variables |  | |  | |  | |  | |  |
| **Age ˂ 43 yrs** (Md) | 0,547 | | 51 | | 56 | | 1,15 | | 0,88 |
| Beighton > 0,0/9 | 0,163 | | 45 | | 69 | | 1,48 | | 0,79 |
| BMI ≥ 33.65 (LRmax) | 0,644 | | 6 | | 97 | | 2,04 | | 0,97 |
| **TME-Abdominals ≥ 72,62 s** (KLf/g) | **0,022** | | 36 | | 86 | | 2,58 | | 0,74 |
| TME-Back ≥ 236,18 s (KLf/g) | 0,078‡ | | 15 | | 97 | | 5,43 | | 0,87 |
| PPT-SitStand ≥ 11,2 s (LRmax) | 0,100 | | 51 | | 67 | | 1,53 | | 0,74 |
| PPT-SitStand ≥ 7,2 s (KLf/g) | 0,063‡ | | 99 | | 8 | | 1,08 | | 0,11 |
| **PPT-Flexion ≥ 9,91 s** (IY) | 0,071 | | 34 | | 83 | | 2,04 | | 0,79 |
| PPT-Flexion ≥ 11,4 s (KLf/g) | 0,078 | | 26 | | 89 | | 2,38 | | 0,83 |
| **PPT-Reach ≥ 0,76/1** (KLf/g) | **0,012** | | 26 | | 94 | | 4,75 | | 0,78 |
| PPT-Reach ≥ 0,67/1 (ROC) | **0,040** | | 43 | | 78 | | 1,95 | | 0,73 |
| PPT-Reach ≥ 0,63/1 (∆MinSnSp) | 0,119 | | 58 | | 58 | | 1,40 | | 0,71 |
| PPT-Reach ≥ 0,80/1 (LRmax) | **0,019**‡ | | 15 | | 99 | | 11,02 | | 0,86 |
| Class-B variables |  | |  | |  | |  | |  |
| Treatment expectation ≥ 4,22 /5 (Md) | 0,069 | | 58 | | 61 | | 1,50 | | 0,68 |
| **FABQ-PA ˂ 12/24** (KLf/g) | **0.029** | | 42 | | 81 | | 2,13 | | 0,73 |
| PCS˂ 23/52 (KLf/g) | 0.113 | | 64 | | 53 | | 1,36 | | 0,68 |
| HPA-leisure≥ 1,75 /5 (LRmax) | 0,153‡ | | 98 | | 11 | | 1,10 | | 0,17 |
| PDIcog ˂ 25 /100 (∆MinSnSp) | 0,076 | | 43 | | 75 | | 1,74 | | 0,75 |
| PDIsoma ˂ 9,1 /100 (LRmax) | 0,078‡ | | 15 | | 97 | | 5,43 | | 0,87 |
| **Illness perception ˂ 44 /80** (Md) | **0,025** | | 55 | | 69 | | 1,79 | | 0,65 |
| **Illness perception ˂ 45 /80** (KLf/g) | **0,007** | | 62 | | 67 | | 1,87 | | 0,57 |
| Class-C variables |  | |  | |  | |  | |  |
| **PSLR-Pain ROM-min ˂ 68°** (KLf/g) | **0,022** | | 85 | | 36 | | 1,33 | | 0,42 |
| **PSLR-Max ROM-Mean ˂ 93°** (KLf/g) | 0,155 | | 87 | | 25 | | 1,16 | | 0,53 |
| PSLR-Max ROM-Min ˂ 52° (KLf/g) | **0,044**‡ | | 17 | | 97 | | 6,11 | | 0,85 |
| LumbLatFlx -Min ≥ 36° (LRmax) | 0,234‡ | | 11 | | 97 | | 4,08 | | 0,91 |
| LumbFlx-ROM ˂ -16° (∆MinSnSp) | 0,058 | | 57 | | 64 | | 1,57 | | 0,68 |
| LumbFlx-ROM ≥ -2° (ROC) | 0,188‡ | | 17 | | 94 | | 3,06 | | 0,88 |

* Only variables that showed a trend (P < 0.20) with Pearson's Chi-square test or had an LR+ > 2 were retained as candidates and presented in this table, with the exception of the model fit variables (age, BMI). Variables retained in the multivariate logistic models are in bold type.

† Dichotomization method: IY: Youden index; KL: Kullback-Leibler distances; LRmax = maximization of likelihood ratio (LR); Med: median; ROC = ROC curve optimum point; ∆MinSnSp: minimum difference between the rate of true positives and the rate of true negatives.

‡ Fisher's exact test for small numbers. P values ≤ 0.05 are in bold type.

‡‡ When a cell in the contingency table was empty (0), 0.5 was added to allow estimation of these statistics (to interpret with caution)

**Table S2-4. Comparisons between success (n = 53) and failure (n = 36) subgroups at time T0 on dichotomous variables - Results for participants used for CPR at T34.**

| Variables* | Pearson’s Chi-square | | Indicators of diagnosis performance | | | |
| --- | --- | --- | --- | --- | --- | --- |
|  | *P* value ‡ | | Se | Sp | LR+ | LR- |
| Class-A variables | |  |  |  |  |  |
| Sex (male) | | 0,800 | 47 | 56 | 1,06 | 0,95 |
| MCIP-HipIR-Pas-max positive | | 0,196 | 53 | 61 | 1,36 | 0,77 |
| **MCIP-HipE-Act-max negative** | | 0,109 | 42 | 75 | 1,66 | 0,78 |
| Abe-Mvt positive | | **0,044** | 40 | 81 | 2,04 | 0,75 |
| Class-B variables | |  |  |  |  |  |
| None | |  |  |  |  |  |
| Class-C variables | |  |  |  |  |  |
| StartBack positive (medium/high risk) | | 0,066 | 47 | 72 | 1,70 | 0,73 |

* Only variables that showed a trend (P < 0.20) with Pearson's Chi-square test or had an LR+ > 2 were retained as candidates and presented in this table, with the exception of the model fit variable (Sex). Variables retained in the multivariate logistic models are in bold type.

‡ P values ≤ 0.05 are in bold type.
