# Supplementary material for: Derivation of clinical prediction rules for identifying patients with non-acute low back pain who respond best to a lumbar stabilization exercise program at post-treatment and six-month follow-up
Source: PLoS One. 2022 Apr 27;17(4):e0265970. doi: 10.1371/journal.pone.0265970 (PMC9045609; doi:10.1371/journal.pone.0265970)
Supplement: S3 File — Logistic regression multivariate models for success at the end of treatment (T8) and at the six-month follow-up (T34) and rational to select the final clinical prediction rule of success (best models) at each time point. (DOCX) [file pone.0265970.s003.docx]

**SUPPORTING FILE 3**

**Clinical prediction rule for success at the end of treatment (T8)**

Nine models (logistic regressions) of success were developed. The four most promising models (models 3, 5, 8, and 9) were selected; those for which (1) all variables reached statistical significance and (2) the model achieved an LR+ greater than the preliminary study success CPR (LR+ = 4.0) of Hicks, Fritz [1]. Table S3-1 shows the variables (or predictors) of the four selected models, their associated beta coefficients, and their rank in terms of relative contribution to each model. Models 1 and 2, although simpler to use (two or three variables), only achieved LR+ of 4.17 and 4.44 respectively and are not presented. Models 3 and 5 consist only of class-A variables while models 8 and 9 also include class-B variables. None of the models include class-C variables.

Table S3-1. Coefficients of the predictive models of success at T8 and their 95% confidence intervals (95% CI).

| Selected variables | Model 3  (4 variables) | Model 5 (5 variables) | Model 8 (6 variables) | Model 9 (7 variables) |
| --- | --- | --- | --- | --- |
|  | B (CI 95%) | B (CI 95%) | B (CI 95%) | B (CI 95%) |
| PPT-Reach ≥ 0,75/1 | 1,78 (0,32 - 3,23) ^3^ | 1,63 (0,095–3,16) ^3^ | 1,86 (0,24 - 3,48) ^5^ | 1,99 (0,30 - 3,67) ^5^ |
| MCIS-HipAR-Pas-max positive | 1,58 (0,59 - 2,57) ^3^ | 1,79 (0,76–2,82) ^4^ | 2,02 (0,92 - 3,12) ^6^ | 2,15 (1,00 - 3,30) ^6^ |
| Abe-Mvt positive | 1,40 (0,30 - 2,50) ^2^ | 1,60 (0,45 - 2,76) ^2^ | 1,55 (0,34 - 2,75) ^4^ | 1,50 (0,28 - 2,72) ^4^ |
| Beighton ˂ 5/9 | 1,65 (0,00 - 3,30) ^1^ | 1,98 (0,45 - 2,76) ^1^ | 2,20 (0,37 - 4,03) ^1^ | 2,45 (0,56 - 4,34) ^2^ |
| TME-Back ≥ 225,56 s |  | 2,33 (0,29 - 3,67) ^1^ | 2,33 (0,10 - 4,55) ^2^ | 2,21 (0,95 - 4,47) ^4^ |
| Illness Perception ≤ 38,5 /80 |  |  | 1,49 (0,19 - 2,78) ^3^ | 1,85 (0,44 - 3,26) ^3^ |
| FABQ-PA ≥ 6,5/24 |  |  |  | 2,03 (0,13 - 3,93) ^1^ |
| Constant | -2,48 | -3,05 | -3,64 | -5,81 |

B: Beta Coefficient (Log odds); ()^n^: Ranking of predictors according to the Adequacy statistic (n = 1 means that it represents the most influential predictor; the same rank can be assigned when this indicator gives the same score). The Adequacy statistic gives the individual explanatory value of the predictor and thus its explanatory strength. It is the ratio of -2log-likelihood (-2LL) of the predictor to -2LL of the complete Model, and represents the proportion of the log-likelihood of the complete Model (considering all the predictors) that is explained by each predictor individually.

The diagnostic performance and fit indicators for the four selected models are presented in Table S3-2. The selected models, although more complex (four to seven variables), perform better (LR+ between 7.91 and 21.7) than the success CPR in the preliminary study (LR+ = 4.0) by Hicks, Fritz [1]. LR+ is the most important statistical indicator for a successful CPR.

Table S3-2. Indicators of diagnostic performance and fit of predictive models at T8 (95% CI).

|  | Model 3 (4 variables) | Model 5 (5 variables) | Model 8 (6 variables) | Model 9 (7 variables) |
| --- | --- | --- | --- | --- |
| ***Indicators of diagnosis performance*** |  |  |  |  |
| - Sensitivity | 35,2 (22,4 - 47,9) | 37,0 (24,2 - 49,9) | 50,0 (36,7 - 63,3) | 48,1 (34,8 - 61,5) |
| - Specificity | 95,6 (89,5 - 100) | 95,6 (89,5 - 100) | 95,6 (89,5 - 100) | 97,8 (93,5 - 100) |
| - Positive likelihood ratio (LR+) | **7,91** (1,95 - 32,2) | **8,33** (2,06 - 33,7) | **11,25** (2,83 - 44,8) | **21,7** (3,1 - 153,5) |
| - Negative likelihood ratio (LR-) | 0,68 (0,55 - 0,83) | 0,66 (0,53 - 0,82) | 0,52 (0,40 - 0,69) | 0,53 (0,41 - 0,69) |
| - Positive predictive value (PV+) | **90,5** (77,9 - 100) | **91,0** (78,9 - 100) | **93,1** (83,9 - 100) | 96,3 (89,2 - 100) |
| - Negative predictive value (PV-) | 55,1 (44,1 - 66,2) | 55,8 (44,7 - 66,9) | 61,4 (50,0 - 72,8) | 61,1 (49,8 - 72,4) |
| - Area under the curve(AUC), P value*** | 0,78 (0,68 - 0,87) | 0,79 (0,71 - 0,88) | 0,83 (0,75 - 0,91) | 0,85 (0,78 - 0,93) |
| - Post-treatment probability of success (%) † | 88 (68-96) | 89 (69-97) | 91 (76-97) | 95 (78-99) |
| ***Explanatory and adjustment indicators*** |  |  |  |  |
| - Nagelkerke’s R^2^ coefficient (%) | 32,2 | 37,8 | 42,9 | 47,5 |
| - Hosmer & Lemeshow Test (χ2, p) | (χ^2^=4,31; *P* = 0,37) | (χ^2^=3,11; *P* = 0,79) | (χ^2^=2,31; *P* = 0,89) | (χ^2^= 2,98; *P* = 0,89) |

* P values are all ˂ 0.001; Hosmer & Lemeshow Test: goodness-of-fit test (must be nonsignificant).

† Estimated with Fagan nomogram (http://araw.mede.uic.edu/cgi-bin/testcalc.pl), knowing LR+, LR-, and estimating prevalence (here 54/110 patients, or 49%). For Model 3, this indicates that the probability of success increases from 49% (without CPR use) to 88% (with CPR use).

Another indicator provides important complementary information, namely the probability of success in post-treatment estimated with the positive predictive value (PV+). This indicator is analogous to what is calculated using the Fagan nomogram, as done in the landmark study by Hicks, Fritz [1]. For comparison purposes, the estimate given by the Fagan nomogram (to be referred to as VP-Fagan) has also been reported in Table S3-2 because it is commonly used in clinical practice. VP-Fagan estimates that using the different success CPRs (model 3: LR+ = 7.91; model 5: LR+ = 8.33; model 8: LR+ = 11.25; model 9 : LR+ = 21.7) would result in a shift from 49% success (without CPR) to much higher post-treatment success of 88% (95% CI: 68-96), 89% (95% CI: 69-97), 91% (95% CI: 76-97), and 95% (95% CI: 78-99), respectively (note that PV+ are slightly higher by 1-2%). On the other hand, to go from a post-treatment probability of 88% (model 3) to 95% (model 9), three variables must be added to the CPR (TME-Back, Illness Perception, and FABQ-PA), which significantly lengthens the clinical assessment for a relatively minor gain (of 7%) in post-treatment success probability. Finally, it is important to note that the specificity of model 3 (95.6%) for the model with an LR+ of 7.91 is almost the same as in model 9 (specificity = 97.8%) with an LR+ of 21.7. Consequently, model 3 in Table S3-1, which includes four class-A variables, is retained as the final CPR of success at T8. Note that the other statistical indicators are also favourable.

**Clinical prediction rule for success at the six-month follow-up (T34)**

Sixteen models (logistic regressions) of success were developed. The six most promising models (criteria stated in the previous section) are described in Table S3-3 (variables and beta coefficients) and Table S3-4 (diagnostic performance and fit indicators). The first two models (models 2 and 3) consist only of class-A variables, the next two (models 8 and 12) of class-A and class-B variables, while the last two (models 14 and 16) also include class-C variables.

On the basis of LR+, there is a significant discrepancy, to the advantage of the last two models (14 and 16), but these models include class-C variables that have no theoretical relationship with lumbar instability. These models were eliminated but will be used as discussion points. Note that the PV+ is only 2-3% higher for these models (95.7 and 95.8%) than for models 3 and 12 (92.3 and 93.8%), which represents a negligible advantage. Note also that the specificity of the six selected models is equivalent (97%).

Among the first four models, model 3 (LR+ = 8.15), with three class-A variables, and model 12 (LR+ = 10.2), with 6 variables (class-A and -B), stand out based on LR+. On the other hand, the PV+ of these two models is similar (92.3 and 93.8% respectively), and the simpler model (model 3 with three variables) would be more practical (lower clinical assessment burden). Model 3 was therefore retained as the final CPR of success at T34.

Table S3-3. Coefficients of the predictive models of success at T34 and their 95% confidence intervals (95% CI).

| Selected variables | Model 2 | Model 3 | Model 8 | Model 12 |
| --- | --- | --- | --- | --- |
|  | B (CI 95%) | B (CI 95%) | B (CI 95%) | B (CI 95%) |
| MCIP-HipE-Act-max negative | 1,05 (0,003-2,09) ^2^ | 1,13 (0,11-2,15) ^1^ | 1,17 (0,055-2,28) ^1^ | 1,22 (0,02-2,42) ^2^ |
| TME-abdominals ≥ 72,62 s | 1,58 (0,38-2,77) ^4^ | 1,31 (0,14-2,48) ^2^ | 1,66 (0,39-2,92) ^3^ | 1,75 (0,44-3,05) ^5^ |
| PPT-flexions ≥ 9,91 s | 1,54 (0,33-2,75) ^3^ |  | 1,74 (0,44-3,03) ^2^ | 2,47 (0,97-3,97) ^3^ |
| Age ˂ 43 yrs | 0,60 (-0,38-1,58) ^1^ |  |  | 0,92 (-0,19-2,03) ^1^ |
| PPT-Reach ≥ 0,76/1 |  | 2,07 (0,45-3,69) ^3^ |  |  |
| Illness perception ˂ 45 /80  Illness perception ˂ 44 /80 |  |  | 1,44 (0,41-2,47) ^4^  / | /  1,46 (0,32-2,61) ^4^ |
| FABQ- PA ˂ 12/24 |  |  |  | 1,44 (0,27-2,6) ^4^ |
| PSLR-Pas-Max ROM - Mean ˂ 93° |  |  |  |  |
| PSLR-Pain ROM - Min ˂ 68° |  |  |  |  |
| Constant | -0,10 | -0,58 | -1,47 | -2,54 |
|  |  |  |  |  |
| Continuation of Table (last 2 models) | Model 14 | Model 16 |  |  |
|  | B (CI 95%) | B (CI 95%) |  |  |
| MCIP-HipE-Act-max negative | 1,18 (0,05-2,31) ^1^ | 1,47 (0,20-2,75) ^1^ |  |  |
| TME-abdominals ≥ 72,62 s | 1,54 (0,23-2,85) ^2^ | 2,58 (0,96-4,20) ^2^ |  |  |
| PPT-flexions ≥ 9,91 s |  | 2,38 (0,83-3,94) ^2^ |  |  |
| Age ˂ 43 ans |  |  |  |  |
| PPT-Reach ≥ 0,76/1 | 2,49 (0,60-4,38) ^3^ |  |  |  |
| Illness perception ˂ 45 /80  Illness perception ˂ 44 /80 | /  1,07 (0,005-2,14) ^2^ | /  1,68 (0,40-2,96) ^3^ |  |  |
| FABQ- PA ˂ 12/24 | 1,84 (0,49-3,20) ^2^ | 2,09 (0,65-3,53) ^3^ |  |  |
| PSLR-Pas-Max ROM - Mean ˂ 93° | 1,80 (0,29-3,32) ^1^ |  |  |  |
| PSLR-Pain ROM - Min ˂ 68° |  | 2,51 (0,86-4,14) ^3^ |  |  |
| Constant | -3,15 | -4,53 |  |  |
|  |  |  |  |  |

B: Beta Coefficient (Log odds); ()^n^: Ranking of predictors according to the Adequacy statistic (n = 1 means that it represents the most influential predictor; the same rank can be assigned when this indicator gives the same score). The Adequacy statistic gives the individual explanatory value of the predictor and thus its explanatory strength. It is the ratio of -2log-likelihood (-2LL) of the predictor to -2LL of the complete Model and represents the proportion of the log-likelihood of the complete Model (considering all the predictors) that is explained by each predictor individually.

Table S3-4. Indicators of diagnostic performance and fit of predictive models at T34 (95% CI).

|  | Model 2 (4 variables) | Model 3 (3 variables) | Model 8 (4 variables) | Model 12 (6 variables) |
| --- | --- | --- | --- | --- |
| ***Indicators of diagnosis performance*** |  |  |  |  |
| - Sensitivity | 20,75 (9,84-31,67) | 22,64 (11,37-33,9) | 15,1 (5,5-24,7) | 28,3 (16,2-40,4) |
| - Specificity | 97,2 (91,8-100) | 97,22 (91,85-100) | 97,2 (91,8-100) | 97,2 (91,8-100) |
| - Positive likelihood ratio (LR+) | **7,47** (1,01-55,37) | **8,15** (1,11-59,9) | **5,43** (,71-41,6) | **10,2** (1,41-73,8) |
| - Negative likelihood ratio (LR-) | 0,82 (0,70-0,95) | 0,80 (0,68-0,93) | 0,87 (0,77-0,99) | 0,74 (0,62-0,88) |
| - Positive predictive value (PV+) | **91,67** (76,0-100) | **92,3** (77,8-100) | **89,0** (68,4-100) | **93,8** (81,9-100) |
| - Negative predictive value (PV-) | 45,45 (34,33-56,57) | 46,1 (34,8-57,3) | 43,8 (32,9-54,6) | 47,9 (36,5-59,4) |
| - Area under the curve (AUC), P value*** | 0,728 (0,62-0,84) | 0,73 (0,62-0,83) | 0,77 (0,67-0,87) | 0,82 (0,73-0,91) |
| - Post-treatment probability of success (%) † | 89 (58-98) | 90 (61-98) | 86 (51-97) | 92 (66-99) |
| ***Explanatory and adjustment indicators*** |  |  |  |  |
| - Nagelkerke’s R^2^ coefficient (%) | 22,0 | 23,4 | 30,3 | 39,5 |
| - Hosmer & Lemeshow Test (χ2, p) | (χ^2^=4,15; *P* =0,66) | (χ^2^=,11; *P* =0,99) | (χ^2^=2,87; *P* =0,90) | (χ^2^=1,84 ; *P* =0,99) |
|  | Model 14  (6 variables) | Model 16  (6 variables) |  |  |
| ***Indicators of diagnosis performance*** |  |  |  |  |
| - Sensitivity | 41,5 (28,2-54,8) | 43,4 (30,0-56,7) |  |  |
| - Specificity | 97,2 (91,8-100) | 97,2 (91,8-100) |  |  |
| - Positive likelihood ratio (LR+) | **14,9** (2,11-106,0) | **15,6** (2,21-110,6) |  |  |
| - Negative likelihood ratio (LR-) | 0,60 (0,48-0,76) | 0,58 (0,46-0,74) |  |  |
| - Positive predictive value (PV+) | **95,7** (87,3-100) | **95,8** (87,8-100) |  |  |
| - Negative predictive value (PV-) | 53,0 (41,0-65,1) | 53,8 (41,7-66,0) |  |  |
| - Area under the curve (AUC), P value*** | 0,83 (0,75-0,92) | 0,86 (0,80-0,93) |  |  |
| - Post-treatment probability of success (%) † | 94 (75-99) | 95 (76-99) |  |  |
| ***Explanatory and adjustment indicators*** |  |  |  |  |
| - Nagelkerke’s R^2^ coefficient (%) | 41,6 | 48,7 |  |  |
| - Hosmer & Lemeshow Test (χ2, p) | (χ^2^=5,55; *P* =0,70) | (χ^2^=4,49; *P* =0,81) |  |  |

* P values are all ˂ 0.001; Hosmer & Lemeshow Test: goodness-of-fit test (must be nonsignificant).

† Estimated with Fagan nomogram (http://araw.mede.uic.edu/cgi-bin/testcalc.pl), knowing LR+, LR-, and estimating prevalence (here 53/100 patients, or 53%). For Model 3, this indicates that the probability of success increases from 53% (without CPR use) to 90% (with CPR use).

REFERENCE

1. Hicks GE, Fritz JM, Delitto A, McGill SM. Preliminary development of a clinical prediction rule for determining which patients with low back pain will respond to a stabilization exercise program. Arch Phys Med Rehabil. 2005;86(9):1753-62.
