# Supplementary material for: Derivation of clinical prediction rules for identifying patients with non-acute low back pain who respond best to a lumbar stabilization exercise program at post-treatment and six-month follow-up
Source: PLoS One. 2022 Apr 27;17(4):e0265970. doi: 10.1371/journal.pone.0265970 (PMC9045609; doi:10.1371/journal.pone.0265970)
Supplement: S4 File — Rationale for the inclusion of class-B and class-C variables in the multivariate logistic models for success following a lumbar stabilization exercise program. (DOCX) [file pone.0265970.s004.docx]

**SUPPORTING FILE 4**

**Rationale for the inclusion of class-B variables in the multivariate logistic models for success following a lumbar stabilization exercise program (LSEP).**

When Class B variables were eligible, in addition to Class A variables, two variables were introduced at both T8 and T34 (**S3** **file**), namely illness perceptions measured with the 8-item B-IPQ [1] and fear-avoidance beliefs associated with physical activity (FABQ-AP) measured with the 5-item subscale of this questionnaire [2]. Like class-C variables, class-B variables are nonspecific to the LSEP but have the potential to be associated with its success through adherence to the home-based exercise program. Multivariate analyses to predict adherence to the home-based LSEP allowed a link to be made with our primary outcome measure (ODI), as reported elsewhere [3]. Because this multivariate model and the study design could not determine the direction and causality of this relationship, both interpretations are possible (ODI predicts adherence or adherence predicts ODI), and a bidirectional causal relationship cannot be ruled out.

Illness perceptions (B-IPQ), with a score ≤ 38.5/80 (models at T8) or ˂ 44/80 or 45/80 (models at T34), would predict the success of the LSEP. Thus, patients with scores higher than these thresholds, corresponding to poor perception of one's health condition, would not benefit as much from LSEP, which could be explained by poor adherence to LSEP done at home. Multivariate analyses of adherence prediction support this hypothesis [3]. Indeed, by far the most influential predictor of adherence was the global rating of change (variable GROC) perceived by the participants and GROC was in turn predicted by B-IPQ as the most influential predictor [3]. The same results were obtained in the preliminary study (n = 48 participants), with the explained variance of adherence and GROC being 50 and 58%, respectively [4]. The present study, including 110 participants, now explains 76 and 50% of the variance of adherence and GROC, demonstrating the robustness of the results [3].

Illness perceptions (or representations) thus indirectly predicts adherence but, unlike GROC, it can be assessed at the initial patient assessment. These representations can be modified by education when patients seek answers [5]. A discussion is needed when patients have difficulty freeing themselves from certain beliefs or fears regarding causes, efficacy, consequences, chronology, or symptoms because these elements represent potential barriers to their adherence to their home exercise program. Representations can also be addressed indirectly. In this case, clinicians must propose strategies that make sense to patients, and help them adopt the behavior and see its impact [5]. Moreover, it appears that representations can change as quickly as in the first two weeks of a program [5]. Physical therapists can also collaborate with other disciplines (e.g., psychologists, occupational therapy, and medicine) to design interventions to improve behavior change toward better adherence to home exercise programs [6].

Fear-avoidance and beliefs about physical activity (FABQ-AP) was introduced at T8 (model 9; score > 6.5 / 24) as well as at T34 in three models, but this time with an opposite interpretation (score ˂ 12 / 24), which is not uncommon in the results of other CPRs, as detailed later. This variable was not retained in our multivariate analyses in relation to adherence to the home-based LSEP [3], so the mechanism of action is possibly different. Surprisingly, the FABQ questionnaire has traditionally been considered in the derivation stage of various CPRs for different physical therapy treatments [7-11], and sometimes without any apparent underlying hypothesis to account for the possible relationship with treatment. One possible consideration is that it may exclude some patients who would be more in need of a psychological than a biomedical intervention.

The FABQ work subscale was retained by Cai, Pua [7] in their CPR for success after spinal traction (cutoff ≤ 21 / 42) and by Flynn, Fritz [8] in their CPR for success after spinal manipulation (cutoff ˂ 19 / 42). These findings suggest that patients with high FABQ-work scores would not respond to these treatments. This fits with underlying biomedical mechanism of action for these treatments. The physical activity subscale (FABQ-AP) was also retained by Cleland, Childs [11] in their CPR for success (threshold ˂ 12 / 24) after thoracic spinal manipulation and by Hicks, Fritz [9] in their CPR for failure (threshold ˂ 9 / 24) after a LSEP. Cleland, Childs [11] were unable to hypothesize an explanation for their results. The interpretation of the results of the present research (at least for the model at T8) is in line with Hicks, Fritz [9] CPR for failure, if the interpretation of Hicks' findings (predicting failure instead of success) is reversed. Thus, the LSEP would primarily help patients with moderate to high scores of the FABQ-AP subscale to overcome their fears, which in turn would improve perceived disability, as proposed by the fear avoidance model [12]. If not through adherence to home exercises, it could be through the performance of exercises done in the clinic. It should be remembered that the LSEP, with its three phases that allow motor and functional challenges to be overcome, offers a gradual exposure that allows fears to be confronted, which are winning ingredients for this type of patient. On the other hand, like the CPR for success after thoracic spinal manipulation [11], the opposite results observed at T34 (score ˂ 12 / 24) suggest that participants must not have too high a level of fear at the beginning of the LSEP to achieve success. The role of FABQ-AP in different CPRs in physical therapy definitely remains to be clarified.

**Rational for the inclusion of class-C variables in the multivariate logistic models for success following a lumbar stabilization exercise program (LSEP).**

Class-C variables are essentially variables measuring the range of motion (ROM) at different joints. There are no studies that have linked mobility to lumbar instability, other than the mobility between two lumbar vertebrae. These variables are therefore non-specific, which means that they could influence the outcome of any exercise program. These variables inform the prognosis of the patient and thus could be considered in "prognostic" CPRs, i.e., regardless of the treatment followed, but can only discredit "prescriptive" CPRs such as those derived in the present study [13]. Indeed, considering the design of the present study (cohort study), i.e., without the consideration of a control group to exclude non-specific clinical changes to the LSEP, it was judged from the outset that class-C variables could not be part of the final CPRs selected from all the studied multivariate models. This strategy would help reduce the likelihood that these final CPRs would be non-specific to LSEP.

The interest of having produced models with class-C variables is to show that they can have predictive value, even after including class-A and B variables in the model, which was the case for the prediction of success at T34 only. From the perspective of deriving a prognostic CPR, this information is therefore of value.

Models 14 and 16 (**S3** **file**) for predicting success at T34 included two variables from the passive straight-leg raise test, the bilateral average ROM (PSLR-Pas-Max ROM - Mean ˂ 93°; model 14) and the between-side minimal ROM when pain occurs (PSLR-Pain ROM - Min ˂ 68°; model 16). These two variables came from the same test and were highly correlated before their dichotomization (Pearson's r = 0.83; P ˂ 0.001), revealing that they capture essentially the same information. The first variable had been retained in the Hicks, Fritz [9] CPR for success after a LSEP, which corresponds to our variable called PSLR-Pas-Max ROM - Mean > 91°, which appears very similar (91° vs 93°) but in the opposite direction. A decrease in this variable is associated with the presence of radiculopathy and poor prognosis, making the results of models 14 and 16 counterintuitive in terms of the interpretation of these variables. However, this test has a high sensitivity to radiculopathy related to disc herniation combined with poor specificity [14]. Given that our participants showing neurological problems were excluded, these findings should not be associated with radiculopathy. This test could simply be an indication of reduced hamstring flexibility and/or reduced sciatic nerve mobility. It has also been associated with a decrease in blood flow to the nerve roots [15], which could, in theory, be related to irritation of the nerve roots adjacent to an unstable lumbar segment [16].

REFERENCES

1. Broadbent E, Petrie KJ, Main J, Weinman J. The brief illness perception questionnaire. J Psychosom Res. 2006;60(6):631-7.

2. Waddell G, Newton M, Henderson I, Somerville D, Main CJ. A fear-avoidance beliefs questionnaire (FABQ) and the role of fear-avoidance beliefs in chronic low back pain and disability. Pain. 1993;52:157-68.

3. Larivière C, Coutu MF, Henry SM, Preuss R, Sullivan MJL, Roy N, et al. Dérivation de règles de prédiction clinique pour dépister les patients ayant une lombalgie non aiguë qui, lors d’un programme d’exercice de stabilisation lombaire, vivraient un succès thérapeutique. Montréal, Canada, 2022, R-1151-fr. Available from: http://www.irsst.qc.ca/publications-et-outils/publication/i/101146

4. L’Heureux J, Coutu M-F, Berbiche D, Larivière C. Adherence to a home exercise programme following a clinical programme for non-acute non-specific low back pain: an exploratory study. European Journal of Physiotherapy. 2019;22(5):299-308. doi: 10.1080/21679169.2019.1617777.

5. Coutu MF, Baril R, Durand MJ, Cote D, Cadieux G. Clinician-patient agreement about the work disability problem of patients having persistent pain: why it matters. J Occup Rehabil. 2013;23(1):82-92. doi: 10.1007/s10926-012-9387-8.

6. Peek K, Sanson-Fisher R, Mackenzie L, Carey M. Interventions to aid patient adherence to physiotherapist prescribed self-management strategies: a systematic review. Physiotherapy. 2016;102(2):127-35. doi: 10.1016/j.physio.2015.10.003.

7. Cai C, Pua YH, Lim KC. A clinical prediction rule for classifying patients with low back pain who demonstrate short-term improvement with mechanical lumbar traction. Eur Spine J. 2009;18(4):554-61.

8. Flynn T, Fritz J, Whitman J, Wainner R, Magel J, Rendeiro D, et al. A clinical prediction rule for classifying patients with low back pain who demonstrate short-term improvement with spinal manipulation. Spine. 2002;27(24):2835-43.

9. Hicks GE, Fritz JM, Delitto A, McGill SM. Preliminary development of a clinical prediction rule for determining which patients with low back pain will respond to a stabilization exercise program. Arch Phys Med Rehabil. 2005;86(9):1753-62.

10. Stolze LR, Allison SC, Childs JD. Derivation of a Preliminary Clinical Prediction Rule for Identifying a Sub-Group of Patients With Low Back Pain Likely to Benefit From Pilates-Based Exercise. J Orthop Sports PhysTher. 2012;42(5):425-36.

11. Cleland JA, Childs JD, Fritz JM, Whitman JM, Eberhart SL. Development of a clinical prediction rule for guiding treatment of a subgroup of patients with neck pain: use of thoracic spine manipulation, exercise, and patient education. Phys Ther. 2007;87(1):9-23.

12. Vlaeyen JWS, Linton SJ. Fear-avoidance and its consequences in chronic musculoskeletal pain: a state of the art. Pain. 2000;85(3):317-32.

13. Haskins R, Rivett DA, Osmotherly PG. Clinical prediction rules in the physiotherapy management of low back pain: a systematic review. Man Ther. 2012;17(1):9-21.

14. Deyo RA, Rainville J, Kent DL. What can the history and physical examination tell us about low back pain? JAMA. 1992;268(6):760-5.

15. Kobayashi S, Shizu N, Suzuki Y, Asai T, Yoshizawa H. Changes in nerve root motion and intraradicular blood flow during an intraoperative straight-leg-raising test. Spine. 2003;28(13):1427-34.

16. Shacklock M. Neurodynamics. Physiotherapy. 1995;81(1):9-16.
